# Temporal trend of anisometropia incidence in Chinese school-aged children before and during the COVID-19 pandemic

**DOI:** 10.3389/fmed.2024.1322402

**Published:** 2024-02-12

**Authors:** Yin Huang, Kunliang Qiu, Yuancun Li, Hongxi Wang, Mingzhi Zhang

**Affiliations:** Department of Ophthalmology, Joint Shantou International Eye Center of Shantou University and The Chinese University of Hong Kong, Shantou University Medical College, Shantou, China

**Keywords:** anisometropia, COVID-19, children, incidence, risk factors

## Abstract

**Objective:**

To analyze and compare the temporal trends in the incidence of anisometropia among Chinese school-aged children both before and during the COVID-19 pandemic, and to investigate the impact of the pandemic on the incidence of anisometropia.

**Methods:**

We conducted a retrospective study comprising six distinct and independent longitudinal cohorts, each including children aged 6 to 13 years who visited the Joint Shantou International Eye Center between January 2010 and December 2021. Children were grouped into cohorts based on the year of their first eye clinic visit: 2010, 2012, 2014, 2016, 2018, or 2020. Only children without anisometropia at initial visits, followed for 18 ± 6 months, were included. The cumulative incidence and risk factors of anisometropia were analyzed using Kaplan–Meier estimation and Cox proportional hazards regression models. Subgroup analyses were performed based on sex, age groups, initial refractive error status, and initial interocular SE difference. Anisometropic children were further categorized into myopic and non-myopic, with subsequent subgroup analyses conducted.

**Results:**

Of 11,235 children were recruited from six cohorts (2010: *n* = 1,366; 2012: *n* = 1,708; 2014: *n* = 1,896; 2016: *n* = 2,354; 2018: *n* = 2,514; 2020: *n* = 1,397), 869 children developed anisometropia during a mean follow-up of 17.5 ± 3.7 months. After adjustment of confounding factors, we found that the risk of anisometropia remained relatively stable before 2020 but significantly increased in the 2020 cohort (adjusted HR 2.93, 95% CI 2.23 to 3.86; *p* < 0.001). This trend persisted in studies of spherical anisometropia (adjusted HR 2.52, 95% CI 1.60 to 3.97; *p* < 0.001) and cylindrical anisometropia (adjusted HR 2.91, 95% CI 1.69 to 3.62; *p* < 0.001). Older age and a greater initial difference in SE between the two eyes were also significantly associated with a higher risk of developing anisometropia (*p* < 0.001). Subgroup analyses consistently showed increased risk in the 2020 cohort.

**Conclusion:**

This study reveals a concerning rise in anisometropia incidence among Chinese school-aged children during the period of the COVID-19 pandemic. These findings highlight the worrisome rise in anisometropia risk during the COVID-19 pandemic and emphasize the importance of early detection and management to safeguard children’s visual health.

## Introduction

Anisometropia, characterized by a significant difference in refractive error between the two eyes, is a condition that can have profound implications for children’s visual function and overall quality of life (1). This ocular condition has been associated with the development of amblyopia, strabismus, and binocular vision disorders, highlighting the importance of understanding its prevalence and associated risk factors (2–4). Despite multiple studies providing information on the ocular structure, demographic factors, and refractive status of individuals with anisometropia (5–9), the etiology and the connection between refractive anisometropia and lifestyle among school-aged children remain incompletely understood (10). Previous research has suggested a potential association between increased near work activities and a higher risk of myopia and anisometropia in children (11, 12). However, existing research on anisometropia has largely relied on cross-sectional studies, and only a limited number of longitudinal studies have explored temporal trends in anisometropia occurrence.

The year 2020 was marked by the outbreak of the coronavirus disease 2019 (COVID-19) pandemic, which had caused unprecedented changes in the lifestyle and behavior of children worldwide (13, 14). Due to lockdown measures and school closures, many children spent more time indoors and engaged in near work activities such as online learning, reading and gaming (15). Recent studies have highlighted the potential impact of the COVID-19 pandemic on various aspects of healthcare (16, 17), including eye care (18–20). Disruptions in access to healthcare services, changes in lifestyle and screen time patterns, and increased psychological stress during the pandemic may have influenced the development of ocular conditions, including anisometropia, among children. However, limited research has specifically examined the association between the COVID-19 pandemic and the incidence of anisometropia in pediatric populations.

Exploring the potential association between the COVID-19 pandemic and anisometropia will offer insights into the impact of this global health crisis on pediatric eye health. Therefore, the objective of this study was to provide a comprehensive description and comparison of the temporal trend in the incidence of anisometropia among a large cohort of Chinese school-aged children before and during the COVID-19 pandemic. Additionally, the study aimed to explore the potential impact of the COVID-19 pandemic on the occurrence of anisometropia. We hypothesized that the 2020 cohort would show a higher risk of anisometropia than the previous cohorts due to the pandemic-related changes in lifestyle and environment.

## Methods

### Study participants

This was a retrospective cohort study for children aged 6 to 13 years who visited Joint Shantou International Eye Center (JSIEC) of Shantou University and the Chinese University of Hong Kong (Shantou, China) from January 2010 to December 2021. Only the medical records of children who underwent cycloplegic refraction and had no anisometropia at their first visit were reviewed. Children were included only if they first visited JSIEC in 2010, 2012, 2014, 2016, 2018, 2020 and followed up for 18 ± 6 months. The study population was therefore classified into six period groups based on the year of their initial visit. Each cohort represents a distinct group of children with no overlap between older and more recent cohorts. Exclusion criteria included any missing data of required variables and presence of previous intraocular surgeries or intraocular pathology. The required data of individuals such as demographic details, cycloplegic refraction and progression data were extracted from the electronic medical records (EMR) database of JSIEC. Due to privacy and ethical considerations, specific health information, including COVID-19 status, was not included in the dataset. The study was approved by the Human Research Ethics Committee of JSIEC (EC20200120(1)-P14). The protocol adhered to the provisions of the Declaration of Helsinki for research.

### Measurement of visual acuity and refraction

Visual acuity, measured with well-lit Snellen tumbling-E vision charts (Shantou City Medical Equipment, Ltd., Shantou, China), was conducted separately for each eye at a distance of 5 m. The nontested eye was covered, and the right eye was tested first. A single optotype of each size was presented, starting at 6/30. If a letter was not identified, testing began 2 lines above, with the child reading all optotypes on the line sequentially. Correct identification of more than half of the letters on a given line determined the acuity level. Cycloplegic refraction was applied by dropping cyclopentolate 1% (Cyclogel; Alcon Laboratories, Fort Worth, TX) and tropicamide 1% (Mydriacyl; Alcon Laboratories) into each eye four times, with a 5-min interval between each drop. After confirmation of pupillary dilation to at least 8 mm, autorefraction (RK-F1 Refractometer/Keratometer; Canon, Inc., Tochigi, Japan) was performed. Following this, an ophthalmologist examined the autorefraction outcomes of each eye and adjusted them as needed. Based on these modified outcomes, spectacles were prescribed. Follow-up visits every 3 or 6 months were recommended for all children.

### Definitions

The spherical equivalent (SE), which was calculated as the addition of the spherical power and half the magnitude of the cylinder power, was used to classify refractive status. Myopia was defined as SE ≤ -0.50 D based on the refractive status of the right eyes, and emmetropia was defined as −0.50 D < SE < +0.50 D, and hyperopia as SE ≥ +0.50 D. The primary outcome of the study was the incidence of anisometropia, defined as a difference in SE greater than or equal to 1.0 D between the two eyes at any follow-up visit. Cylindrical anisometropia was characterized by a substantial difference in diopter of the cylindrical power ≥ 1.0 D between the eyes, and spherical anisometropia by a substantial difference in diopter of the spherical power ≥ 1.0 D (21). The differences in the angle of the cylindrical refractive error were not taken into account in this study.

### Statistical analysis

Descriptive statistics were employed to summarize the characteristics of the children. Continuous variables were presented as means (standard deviation [SD]) and medians (interquartile range [IQR]), while categorical variables were reported as frequency counts with percentages. One-way analysis of variance (ANOVA) was used to compare the means of continuous variables, and Pearson’s χ^2^ tests were used to compare distributions of categorical variables. The Kaplan–Meier estimation method was used to depict cumulative incidence curves of anisometropia over time for the entire dataset and for children in different time periods. The table of the number at risk was generated to show the number of children at risk of developing anisometropia at different time intervals during follow-up. The log-rank test was used to assess the statistical significance of the differences between the groups. Multivariate Cox proportional hazards regression models were employed to determine the risk factors associated with the development of anisometropia. Hazard ratios (HRs) with corresponding 95% confidence intervals were calculated to measure the strength of association between each risk factor and the development of anisometropia. The models were adjusted for age, sex, baseline SE, and initial interocular SE difference. The proportional hazard (PH) assumption for the Cox proportional hazard model was tested using the Schoenfeld residuals test. Adjusted survival curves for the risk of incident anisometropia in different time periods were plotted. All statistical analyses were performed using R software (version 4.3.0, R Foundation for Statistical Computing, Vienna, Austria).^1^ A two-sided *p* value of <0.05 was considered statistically significant.

### Subgroup analyses

To explore the potential heterogeneity of the effect of time period on the risk of developing anisometropia across different subgroups of children, stratified analyses were conducted based on sex (female and male), age groups (6 to 8 years old, 9 to 11 years old and 12 to 13 years old), initial refractive error status (myopia, emmetropia and hyperopia), and interocular refractive difference at baseline (<0.25 D, 0.25–0.5 D and ≥ 0.5 D). Additionally, for a more comprehensive insight into anisometropia subtypes, children with anisometropia were categorized into myopic anisometropia and non-myopic anisometropia, based on the presence or absence of myopia (SE ≤ −0.50 D in either eye) when anisometropia occurred, and then subgroup analysis was performed in these two groups. Adjusted survival curves were plotted for each subgroup to visualize the risk of incident anisometropia. Multivariate Cox proportional hazards regression models were applied to assess the significance of the associations within each subgroup.

## Results

### Baseline characteristics

A total of 11,235 children who did not have anisometropia (a difference in SE less than 1.0 D) at baseline and completed 1–2 years of follow-up at JSIEC from 2010 to 2021 were enrolled in the current analysis. Among these cases, 5,909 (52.6%) were male, and 5,326 (47.4%) were female, with a mean age of 9.14 ± 2.18 (range: 6–13) years. The average follow-up duration was 17.5 ± 3.7 months (range: 12 to 24 months). Table 1 showed the baseline characteristics of the study population stratified by different time periods. There was no statistically significant difference in sex distribution at baseline between time periods (*p* = 0.89). However, significant differences were observed in the distribution of age, SE and interocular SE difference at baseline (all *p* < 0.001).

**Table 1:** The baseline characteristics of the participants in different period groups.

Baseline characteristic	Overall	2010	2012	2014	2016	2018	2020	Value of p^*^
N	11,235	1,366	1,708	1,896	2,354	2,514	1,397	
Sex, No. (%)								0.89
Female	5,326.0 (47.4%)	657.0 (48.1%)	789.0 (46.2%)	911.0 (48.0%)	1,110.0 (47.2%)	1,198.0 (47.7%)	661.0 (47.3%)	
Male	5,909.0 (52.6%)	709.0 (51.9%)	919.0 (53.8%)	985.0 (52.0%)	1,244.0 (52.8%)	1,316.0 (52.3%)	736.0 (52.7%)	
Age, years								<0.001
Mean (SD)	9.14 (2.18)	9.67 (2.15)	9.50 (2.21)	9.32 (2.19)	9.10 (2.15)	8.83 (2.15)	8.59 (2.07)	
Median (IQR)	9.00 (7.00, 11.00)	10.00 (8.00, 11.00)	10.00 (8.00, 11.00)	9.00 (7.00, 11.00)	9.00 (7.00, 11.00)	9.00 (7.00, 10.00)	8.00 (7.00, 10.00)	
Range	6.00, 13.00	6.00, 13.00	6.00, 13.00	6.00, 13.00	6.00, 13.00	6.00, 13.00	6.00, 13.00	
SE, D								<0.001
Mean (SD)	−0.88 (2.18)	−1.12 (2.29)	−1.03 (2.03)	−1.00 (2.14)	−0.91 (2.23)	−0.73 (2.16)	−0.56 (2.16)	
Median (IQR)	−1.00 (−2.00, 0.00)	−1.25 (−2.50, 0.00)	−1.25 (−2.00, −0.25)	−1.25 (−2.13, −0.25)	−1.13 (−2.13, 0.00)	−0.88 (−2.00, 0.25)	−0.75 (−1.75, 0.38)	
Range	−13.00, 9.75	−9.50, 8.88	−9.25, 9.75	−10.38, 9.63	−10.75, 9.00	−13.00, 9.75	−8.75, 9.50	
Interocular SE difference (absolute), D								<0.001
Mean (SD)	0.28 (0.23)	0.26 (0.22)	0.27 (0.23)	0.27 (0.23)	0.28 (0.23)	0.28 (0.23)	0.31 (0.24)	
Median (IQR)	0.25 (0.13, 0.50)	0.25 (0.00, 0.38)	0.25 (0.13, 0.50)	0.25 (0.13, 0.50)	0.25 (0.13, 0.50)	0.25 (0.13, 0.50)	0.25 (0.13, 0.50)	
Range	0.00, 0.88	0.00, 0.88	0.00, 0.88	0.00, 0.88	0.00, 0.88	0.00, 0.88	0.00, 0.88	
Incidence rate of anisometropia, No. (%)	869 (7.73%)	104 (7.61%)	108 (6.32%)	146 (7.70%)	194 (8.24%)	197 (7.84%)	120 (8.59%)	0.21

D, diopters; SD, standard deviation; IQR, interquartile range. *One-way analysis of variance (ANOVA) was used to compare the means of continuous variables, and Pearson’s χ^2^ tests were used to compare distributions of categorical variables.

### Cumulative incidence analysis of anisometropia

**Figure 1:**
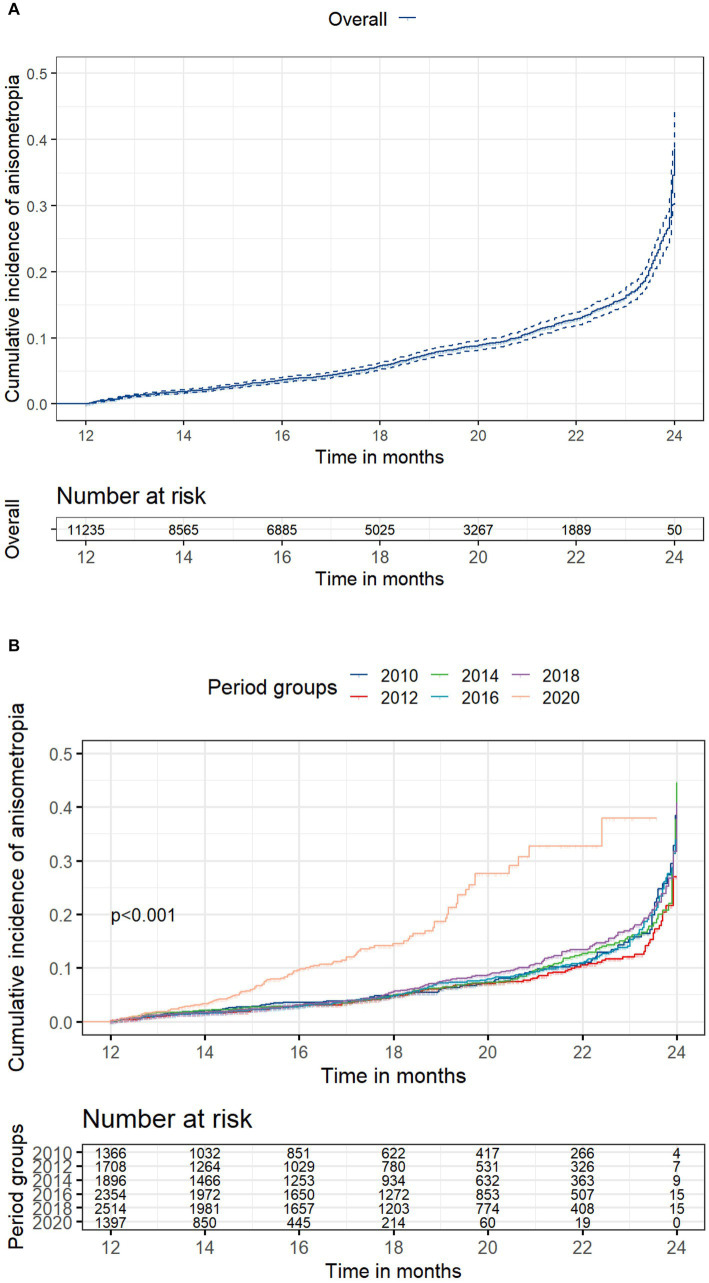
Cumulative incidence of anisometropia. **(A)** Overall cumulative incidence of anisometropia, along with a 95% confidence interval (CI); **(B)** Cumulative incidence of anisometropia stratified by time period cohorts. The number at risk table illustrates the count of children at risk of developing anisometropia at specific time intervals during follow-up. Supplementary Table S1 provides additional details on the number of individuals developing anisometropia and those who were censored at specific time points. Statistical significance between groups was assessed using the log-rank test.

Among the 11,235 children who did not have anisometropia at baseline, 869 (7.73%) developed anisometropia during a mean follow-up of 17.5 ± 3.7 months (range: 12 to 24 months). Table 1 presents the incidence rate of anisometropia in each cohort without considering the impact of follow-up duration. While the 2020 cohort had a higher incidence, the difference was not statistically significant (*p* = 0.21). Figure 1A presents the cumulative incidence of anisometropia over time for entire dataset, along with a 95% confidence interval (CI). Figure 1B displays the cumulative incidence of anisometropia over time for children in different time periods. The number at risk table demonstrates the number of children at risk of developing anisometropia at given time intervals during follow-up. Supplementary Table S1 provides additional details on the number of individuals developing anisometropia and those who were censored (no longer under observation) at specific time points for reasons other than experiencing the event. The log-rank test revealed a significant difference in the incidence of anisometropia between the 2020 cohort and the previous decades (*p* < 0.001).

### Multivariate cox proportional hazards regress ion models

**Figure 2:**
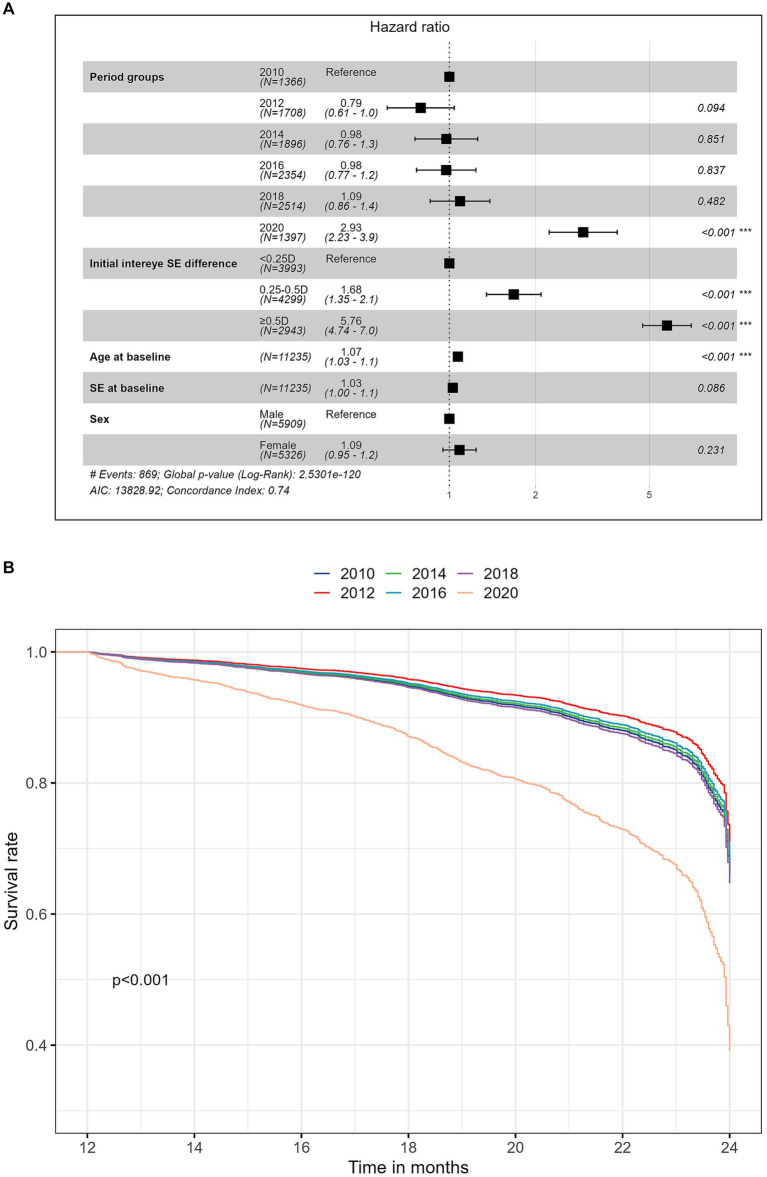
**(A)** Forest plot of the multivariate Cox analysis for the risk of developing anisometropia; **(B)** Adjusted survival curves over time for the risk of incident anisometropia stratified by time period cohorts. The plot was based on Cox proportional hazards regression models, adjusted for age, sex, baseline spherical equivalent (SE), and initial interocular SE difference.

Figure 2A shows the results of multivariate Cox proportional hazards regression models for the risk of developing anisometropia. Figure 2B shows the adjusted survival curves for the risk of incident anisometropia by different time periods. The plot was based on Cox proportional hazards regression models, adjusted for age, sex, baseline spherical equivalent (SE) and initial interocular SE difference. The analysis revealed that the risks of developing anisometropia were similar across different time periods before the 2020 cohort (*p* > 0.05). However, the 2020 cohort had a significantly higher risk of developing anisometropia compared to the previous decades, with an adjusted hazard ratio of 2.93 (95% CI 2.23 to 3.86; *p* < 0.001), indicating over a twofold increased likelihood of anisometropia compared to earlier decades. This trend persisted in studies of spherical anisometropia (adjusted HR 2.52, 95% CI 1.60 to 3.97; *p* < 0.001) and cylindrical anisometropia (adjusted HR 2.91, 95% CI 1.69 to 3.62; *p* < 0.001; Supplementary Table S2). Older age (adjusted HR 1.07, 95% CI 1.03 to 1.11; *p* < 0.001) and a greater initial difference in spherical equivalent between the two eyes (adjusted HR 5.76, 95% CI 4.74 to 7.00; *p* < 0.001) were also significantly associated with a higher risk of developing anisometropia (Figure 2A). However, there was no significant correlation between the incidence of anisometropia and sex or baseline SE in the Cox model (*p* = 0.23).

### Subgroup analyses

**Figure 3:**
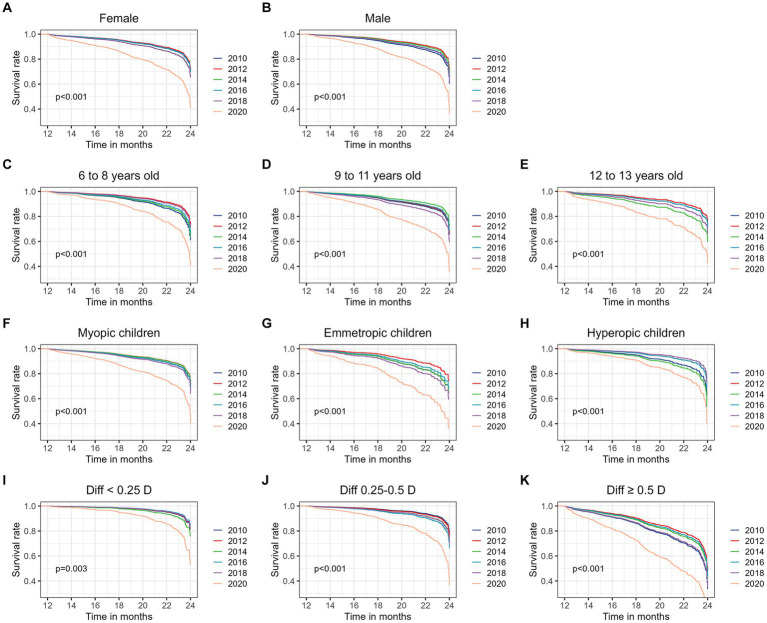
Subgroup survival analysis for the risk of incident anisometropia by different time periods. Adjusted survival curves were plotted for each subgroup to visualize the risk of incident anisometropia. Subgroups were stratified as follows: **(A,B)** Sex Groups—Female and Male; **(C–E)** Age Groups—6 to 8 years old, 9 to 11 years old, and 12 to 13 years old; **(F–H)** Initial Refractive Error Groups—Myopic children, Emmetropic children, and Hyperopic children; **(I–K)** Initial Interocular SE Difference Groups—Diff <0.25 D, 0.25–0.5 D, and ≥ 0.5 D.

**Figure 4:**
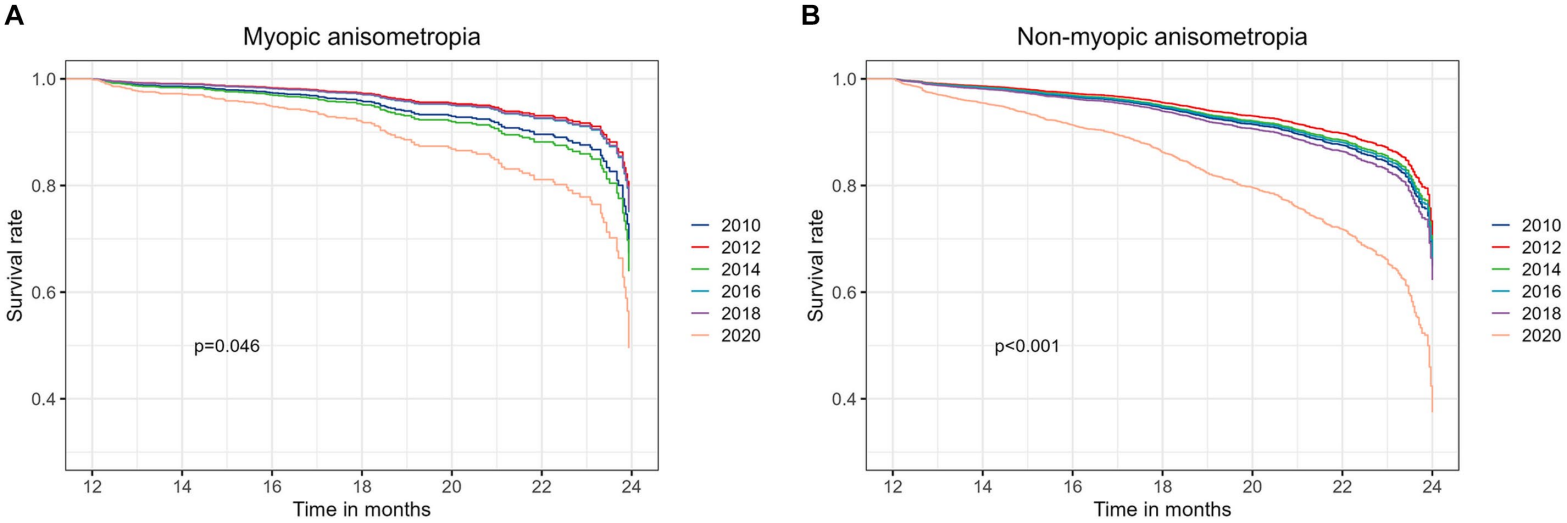
The outcomes of subgroup analyses stratified by myopic anisometropia **(A)** and non-myopic anisometropia **(B)**.

The outcomes of subgroup analyses stratified by sex, age groups (6 to 8 years old, 9 to 11 years old and 12 to 13 years old), initial refractive error status (myopia, emmetropia and hyperopia), and initial interocular difference in SE (Diff <0.25 D, 0.25–0.5 D and ≥ 0.5 D), are presented in Figure 3. Across all examined subgroups, a consistent and noteworthy pattern emerged: the 2020 cohort exhibited a significantly higher risk of developing anisometropia compared to the previous cohorts. Statistical analysis underscored the robustness of this finding, with all value of ps being lower than 0.001, except for the subgroup shown in Figure 3I (*p* = 0.003). The outcomes of subgroup analyses stratified by myopic anisometropia and non-myopic anisometropia are presented in Figure 4. Similarly, irrespective of the anisometropia subtype, a consistent and elevated risk of developing anisometropia was observed in the 2020 cohort when compared to the cohorts from previous years (*p* < 0.05).

## Discussion

In this retrospective cohort study, we investigated the temporal trends of anisometropia incidence in different cohorts of children based on the year of their initial visit, with a particular focus on the potential impact of the COVID-19 pandemic. To the best of our knowledge, this is the first study to explore the association between the COVID-19 pandemic and anisometropia in pediatric populations.

One of the main contributions of this study is the analysis of temporal trends in the incidence of anisometropia over a long period. Our study included a large sample size of 11,235 children who initially did not have anisometropia and were followed up for a duration of 1–2 years. The cumulative incidence curves were plotted to capture the dynamic changes in the incidence anisometropia over time, and the Multivariate Cox proportional hazards regression models were used to identify potential risk factors associated with its development. We found an overall incidence rate of anisometropia of 7.73% in the study population, with a mean follow-up duration of 17.5 months. This finding is consistent with the result reported in a longitudinal study conducted in Singapore, which revealed a 3-year cumulative incidence rate of 7.55% among children aged 7 to 9 years (22). Previous studies have examined the prevalence of anisometropia in school-age children, providing valuable insights into the occurrence of this condition. In a large-scale population-based study in Taiwan, the prevalence of anisometropia among 8-year-old students was found to be 5.3% (12). Another study conducted in an urban area of China, reported a prevalence of 25.6% among schoolchildren aged 7 to 19 years (23). In contrast, a study in Australia found a lower prevalence rate of 1.6% among 6-year-old children (24). However, the prevalence of anisometropia varied across studies due to the differences in ethnicity, age, areas of residence, and study design, making it challenging to directly compare trends over time. By categorizing the study population into different cohorts according to the year of their first visit, we were able to observe the temporal trends of anisometropia incidence. The most striking finding of our study was that the risk of children developing anisometropia was relatively stable across the earlier decades but increased significantly in the 2020 cohort. The children in the 2020 cohort had a more than two-fold higher risk of developing anisometropia than those in the earlier cohorts, after adjusting for age, sex, baseline SE and initial interocular SE difference. This finding was consistent across different subgroups of children based on sex, age, initial refractive status and interocular refractive difference. These results support our hypothesis that the COVID-19 pandemic may have affected the development of anisometropia in children.

The specific mechanisms underlying the association between the COVID-19 pandemic and anisometropia remain unclear. However, several hypotheses can be proposed to explain the heightened risk of anisometropia development in the 2020 cohort. One possible explanation is the increased amount and duration of near work activities during the pandemic-induced lockdown measures and school closures among children, such as online learning, reading, and gaming (25). Previous studies have suggested that extensive near work may induce transient myopia and increase the axial length of the eye, resulting in a mismatch between the refractive power and axial length of the two eyes (26–28). Hu et al. conducted a large-scale cross-sectional school-based study in Eastern China and found that the prevalence and the amount of refractive anisometropia increased with more time spent indoors reading or writing (11). Near work activities typically involve the convergence and accommodation of the eyes to achieve clear and single binocular vision of nearby objects. Mechanical pathways associated with convergence and accommodation are believed to contribute to the development of asymmetrical myopia (10). Previous research has indicated that convergence can exert mechanical forces on the eye, potentially causing transient changes in axial length, especially in downward gaze (29). Accommodation may also influence the development of anisometropia due to ciliary muscle contraction (30–32). Except for the mechanical mechanisms, optical factors such as astigmatism, higher-order aberrations and choroidal response may also affect the quality of retinal image and the visual feedback for ocular growth. Studies have provided evidence of interocular asymmetry in these factors among individuals with anisometropia, potentially contributing to the condition (33–35). In summary, near work activities may play a potential role in the development of anisometropia through mechanical or optical mechanisms. However, the evidence remains inconclusive, and further studies are necessary to establish a causal relationship. Our study contributes to the growing body of evidence by demonstrating a temporal association between the COVID-19 pandemic and an increased incidence of anisometropia in children.

Another possible explanation is that the pandemic may have reduced the outdoor exposure and physical activity of children (16, 17), which may have affected their ocular development and refractive error. Several studies have reported that outdoor exposure and physical activity may have protective effects against myopia and anisometropia by stimulating dopamine release, reducing choroidal thickness and enhancing retinal image quality. The psychological and emotional stress experienced during the pandemic could also be a factor contributing to the increased incidence of anisometropia (36). Stress has been linked to visual disturbances and changes in accommodative function (37), which could potentially affect the development of anisometropia. Understanding the relationship between psychological stress, visual function, and the risk of anisometropia could provide additional insights into this phenomenon.

While the exact mechanisms underlying the observed trends remain to be elucidated, evidence from existing literature suggests that the relationship between the COVID-19 pandemic and anisometropia is often associated with myopic anisometropia (10). However, our study adds a significant nuance to this understanding by indicating that the association extends beyond myopic anisometropia to encompass all types of anisometropia. To explore this further, we conducted subgroup analyses, stratifying participants into myopic anisometropia and non-myopic anisometropia subtypes. As illustrated in Figure 4, our results reveal a consistent and elevated risk of developing anisometropia in the 2020 cohort, regardless of the anisometropia subtype. This observation suggests that the impact of the COVID-19 pandemic on anisometropia incidence is not confined to myopic anisometropia alone but extends to the broader spectrum of anisometropia subtypes. These findings highlight the complex interplay of various factors during the pandemic, including potential changes in near work activities, reduced outdoor exposure, and increased psychological stress. While our study does not conclusively identify the specific mechanisms responsible for the observed increase in anisometropia incidence, it underscores the importance of considering the multifaceted influences of the pandemic on ocular health.

The other findings of this study were that older age and a greater initial difference in SE between the two eyes were significantly associated with a higher risk of anisometropia development. These findings are consistent with previous studies that reported similar risk factors for anisometropia (23, 38). The biological mechanisms underlying these associations are not yet fully understood, but they may involve genetic factors, environmental factors or both. It is possible that older children have more accumulated exposure to environmental factors that affect their ocular growth and refractive error, such as near work, outdoor exposure and eye care services. Additionally, older children may have a greater genetic predisposition to develop anisometropia due to inherited ocular biometry or refractive error. Moreover, it is possible that a greater initial difference in SE between the two eyes may reflect a pre-existing asymmetry in ocular growth or refractive error between the two eyes, which may increase the likelihood of developing anisometropia over time.

The strengths of this study include its large sample size, long follow-up period, standardized measurement methods and comprehensive analysis. However, some limitations should also be acknowledged. First, this study only included children who visited a tertiary eye center in China, which may limit the generalizability of the findings to other populations or settings. Second, this study did not collect data on some potential confounding or mediating factors that may affect the development of anisometropia, such as near work time, outdoor exposure time, physical activity level, psychological stress level. Therefore, we could not directly assess the causal relationship between these factors and the development of anisometropia. Third, this study did not measure other aspects of ocular biometry or visual function that may be related to anisometropia, limiting the assessment of their impact and potential mechanisms involved. Longitudinal studies with detailed information on lifestyle changes, screen time exposure, access to eye care services, and psychological factors during the pandemic would be valuable in elucidating the underlying mechanisms driving the increased incidence of anisometropia. Fourth, a limitation of this study is that the definition of refractive anisometropia did not consider the differences in the angle of the cylindrical refractive error. Future research could benefit from using power vector coordinates to examine the effects of refractive anisometropia on visual function and quality of life, especially for those with high or oblique astigmatism.

In conclusion, this study highlights the increased incidence of anisometropia during the COVID-19 pandemic, suggesting that the COVID-19 pandemic may have had a negative impact on the development of anisometropia among children. This finding has important implications for the prevention and management of anisometropia and its associated complications in pediatric populations, especially during the pandemic. Further research is necessary to confirm and elucidate the association between the COVID-19 pandemic and anisometropia, as well as to explore the underlying mechanisms and potential interventions.

## Data availability statement

The raw data supporting the conclusions of this article will be made available by the authors, without undue reservation.

## Ethics statement

The studies involving humans were approved by the Ethics Committee of Joint Shantou International Eye Center (JSIEC) of Shantou University and the Chinese University of Hong Kong (Shantou city, China). The studies were conducted in accordance with the local legislation and institutional requirements. The Ethics Committee/institutional review board waived the requirement of written informed consent for participation from the participants or the participants’ legal guardians/next of kin because this study was retrospective, informed consent for inclusion was waived.

## Author contributions

YH: Conceptualization, Data curation, Formal analysis, Methodology, Visualization, Writing – original draft, Writing – review & editing. KQ: Supervision, Writing – review & editing. YL: Methodology, Writing – review & editing. HW: Writing – review & editing. MZ: Funding acquisition, Methodology, Supervision, Writing – review & editing.

## Footnotes

^1^ www.R-project.org
