# Supplementary material for: Temporal trend of anisometropia incidence in Chinese school-aged children before and during the COVID-19 pandemic
Source: Front Med (Lausanne). 2024 Feb 12;11:1322402. doi: 10.3389/fmed.2024.1322402 (PMC10894982; doi:10.3389/fmed.2024.1322402)
Supplement: Supplementary file 1 [file Table_1.docx]

Supplementary Material

**Table S1: Additional Details on the Number at Risk Table in the Survival Analysis of the Incidence of Anisometropia in Different Time Periods**

| **Time (Months)** | **12** | **14** | **16** | **18** | **20** | **22** | **24** |
| --- | --- | --- | --- | --- | --- | --- | --- |
| **2010 cohort** |  |  |  |  |  |  |  |
| Number at Risk | 1366 | 1032 | 851 | 622 | 417 | 266 | 4 |
| Events | 0 | 23 | 16 | 10 | 13 | 13 | 29 |
| Censored | 12 | 299 | 165 | 219 | 192 | 138 | 237 |
| **2012 cohort** |  |  |  |  |  |  |  |
| Number at Risk | 1708 | 1264 | 1029 | 780 | 531 | 326 | 7 |
| Events | 2 | 20 | 17 | 16 | 17 | 15 | 21 |
| Censored | 22 | 400 | 218 | 233 | 232 | 190 | 305 |
| **2014 cohort** |  |  |  |  |  |  |  |
| Number at Risk | 1896 | 1466 | 1253 | 934 | 632 | 363 | 9 |
| Events | 0 | 35 | 12 | 21 | 21 | 29 | 28 |
| Censored | 15 | 380 | 201 | 298 | 281 | 240 | 335 |
| **2016 cohort** |  |  |  |  |  |  |  |
| Number at Risk | 2354 | 1972 | 1650 | 1272 | 853 | 507 | 15 |
| Events | 0 | 32 | 24 | 31 | 36 | 22 | 49 |
| Censored | 11 | 339 | 298 | 347 | 383 | 324 | 458 |
| **2018 cohort** |  |  |  |  |  |  |  |
| Number at Risk | 2514 | 1981 | 1657 | 1203 | 774 | 408 | 15 |
| Events | 2 | 37 | 24 | 38 | 32 | 31 | 33 |
| Censored | 15 | 479 | 300 | 416 | 397 | 335 | 375 |
| **2020 cohort** |  |  |  |  |  |  |  |
| Number at Risk | 1397 | 850 | 445 | 214 | 60 | 19 | NA |
| Events | 1 | 36 | 42 | 18 | 19 | 3 | NA |
| Censored | 21 | 489 | 363 | 213 | 135 | 38 | NA |

Number at Risk: the number of individuals still at risk (under observation) at the beginning of each time period.

Events: the number of individuals who developed anisometropia during the respective time period.

Censored: the number of individuals who were censored (no longer under observation) at those specific time points for reasons other than experiencing the event.

NA: not available.

**Table S2: Results of Multivariate Cox Proportional Hazards Regression Models for Spherical and Cylindrical Anisometropia**

| **Covariate** | **HR** | **95% CI (Lower, Upper)** | **p-value** |
| --- | --- | --- | --- |
| **Spherical Anisometropia** |  |  |  |
| **Period groups** |  |  |  |
| 2010 | Reference | - | - |
| 2012 | 0.97 | 0.61, 1.55 | 0.89 |
| 2014 | 1.13 | 0.72, 1.77 | 0.59 |
| 2016 | 0.99 | 0.65, 1.50 | 0.95 |
| 2018 | 1.23 | 0.83, 1.84 | 0.31 |
| 2020 | 2.52 | 1.60, 3.97 | <0.001 |
| **Initial intereye SE difference** | 2.82 | 1.85, 4.31 | <0.001 |
| **Age at baseline** | 1.04 | 0.98, 1.11 | 0.19 |
| **SE at baseline** | 1 | 0.95, 1.05 | 0.92 |
| **Sex** |  |  |  |
| Female | Reference | - | - |
| Male | 0.95 | 0.77, 1.17 | 0.63 |
| **Cylindrical Anisometropia** |  |  |  |
| **Period groups** |  |  |  |
| 2010 | Reference | - | - |
| 2012 | 0.99 | 0.67, 1.47 | 0.96 |
| 2014 | 1.11 | 0.76, 1.62 | 0.59 |
| 2016 | 1.16 | 0.82, 1.65 | 0.39 |
| 2018 | 1.13 | 0.80, 1.59 | 0.49 |
| 2020 | 2.48 | 1.69, 3.62 | <0.001 |
| **Initial intereye SE difference** | 1.94 | 1.35, 2.80 | <0.001 |
| **Age at baseline** | 1.02 | 0.97, 1.07 | 0.53 |
| **SE at baseline** | 0.84 | 0.80, 0.88 | 0.00 |
| **Sex** |  |  |  |
| Female | Reference | - | - |
| Male | 1.04 | 0.88, 1.25 | 0.63 |

HR: Hazard Ratios.

95% CI: 95% Confidence Intervals.
